# Can Government Be Self-Organized? A Mathematical Model of the Collective Social Organization of Ancient Teotihuacan, Central Mexico

**DOI:** 10.1371/journal.pone.0109966

**Published:** 2014-10-10

**Authors:** Tom Froese, Carlos Gershenson, Linda R. Manzanilla

**Affiliations:** 1 Instituto de Investigaciones en Matemáticas Aplicadas y en Sistemas, Universidad Nacional Autónoma de México, Ciudad Universitaria, Mexico City, Distrito Federal, Mexico; 2 Centro de Ciencias de la Complejidad, Universidad Nacional Autónoma de México, Ciudad Universitaria, Mexico City, Distrito Federal, Mexico; 3 Instituto de Investigaciones Antropológicas, Universidad Nacional Autónoma de México, Ciudad Universitaria, Mexico City, Distrito Federal, Mexico; University of Vermont, United States of America

## Abstract

Teotihuacan was the first urban civilization of Mesoamerica and one of the largest of the ancient world. Following a tradition in archaeology to equate social complexity with centralized hierarchy, it is widely believed that the city’s origin and growth was controlled by a lineage of powerful individuals. However, much data is indicative of a government of co-rulers, and artistic traditions expressed an egalitarian ideology. Yet this alternative keeps being marginalized because the problems of collective action make it difficult to conceive how such a coalition could have functioned in principle. We therefore devised a mathematical model of the city’s hypothetical network of representatives as a formal proof of concept that widespread cooperation was realizable in a fully distributed manner. In the model, decisions become self-organized into globally optimal configurations even though local representatives behave and modify their relations in a rational and selfish manner. This self-optimization crucially depends on occasional communal interruptions of normal activity, and it is impeded when sections of the network are too independent. We relate these insights to theories about community-wide rituals at Teotihuacan and the city’s eventual disintegration.

## Introduction

**Figure 1:**
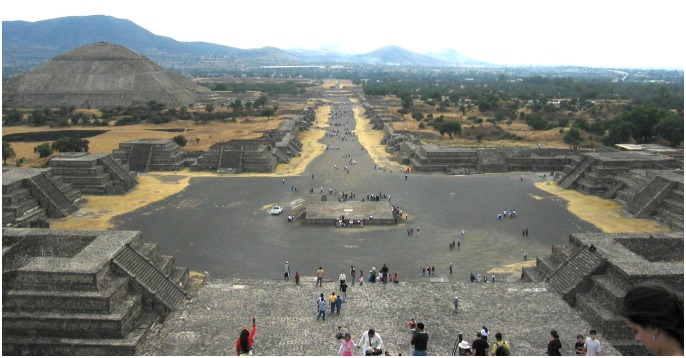
View of the extensive ceremonial center of Teotihuacan as seen from the top of the Pyramid of the Moon. In front is the ceremonial plaza of the Pyramid of the Moon with a small ritual platform in the middle. The Street of the Dead can be seen stretching into the distance, its sides lined with pyramidal platforms that would have originally supported temple structures. Mimicking the mountainous horizon on the left is the Pyramid of the Sun. (Photo courtesy of Iliana Mendoza).

Teotihuacan was a metropolis in the Valley of Mexico. Starting around 100 AD, it rapidly grew into the largest city of Mesoamerica of the first millennium. High-resolution chronology of the city’s center places its collapse around 550 AD [1]. At its peak Teotihuacan was one of the largest population centers of the world. Population estimates vary greatly, but a conservative estimate is that by 150 AD the city’s population had reached a plateau of between 80,000 and 100,000 inhabitants [2]. It covered an area of about 20 km^2^ and was divided into quadrants by its north-south (“Street of the Dead”) and east-west axes (“East Avenue” and “West Avenue”) [3]. In the beginning construction focused on the monumental architecture along the Street of the Dead, in particular on the “Pyramid of the Sun”, the “Pyramid of the Moon”, and the “Feathered Serpent Pyramid” (Figure 1). Later, beginning around 200 AD, more than 2000 large-scale residential units were built to accommodate most of the population, including all socioeconomic statuses [4]. These were solid, roofed structures with open patios and drainage. Each of the so-called “apartment compounds” was shared by a number of households. It is difficult to assess demography in archaeology, and Teotihuacan is no exception. For example, excavation of the Oztoyahualco 15B apartment compound revealed that it contained three nuclear households [4], but we do not know how many members each of them had, perhaps five to ten. More generally, Cowgill [5] estimates that the mean number of domestic units per compound was between three and five, and that on average there were between five and twelve persons in a unit.

No previous or contemporary population center in Mesoamerica was as urbanized as Teotihuacan [6]. Given that it was so unlike the centers that came before, it is an interesting question how we should account for this qualitative transition in social organization. Parsons has formulated the key problem of the city’s origins as follows: “we have to deal with the organizational mechanisms whereby Early Classic (and even latest Formative) Teotihuacan pulled in and (or) attracted large masses of people from considerable distances and coordinated them, and probably much of the whole of central Mexico, into an effective urban system which was adaptive for over 500 years” ([7]: 876). We can divide this problem of origins into two aspects: how to explain the large-scale relocation of whole communities into the city in a very short timespan, and how to explain the coordination of all these diverse people in a way that allowed the city to function in an adaptive manner. Unfortunately, not much direct information about Teotihuacan’s social organization is available, especially about the city’s initial centuries. There is a consensus that there probably was a collective form of government during the later periods [8], because during that time ideology deemphasized individual status and personal achievement in favor of highlighting universally applicable social and cosmological principles [9]. However, opinions diverge about the city’s social organization during the earliest periods. Two competing frameworks of interpretation have been put forward.

On the one hand, there are those who argue that Teotihuacan was centrally controlled by powerful rulers and military elites, who imposed law and order on the inhabitants of the city and conquered distant regions to ensure a constant flow of tribute and sacrificial victims [10]–[12]. On the other hand, there are those who argue that the social organization of the city is more accurately characterized as a decentralized network of diverse semi-autonomous communities that were governed in a corporate manner, and who were more interested in ritual and trade than empire building [13]–[15]. We will briefly indicate some of the key arguments of both approaches.

### Evidence for Centralized Government

Teotihuacan was long viewed as a peaceful theocratic state, but this view has fallen out of favor in recent decades. In line with a growing emphasis of the role of war for state formation, including for the first Mesoamerican states [16], [17], instead there is an emphasis on militarism ([12]: 105–118) and political power [10], [11]. For example, many archaeologists interpret the construction of the Feathered Serpent Pyramid around 200 AD, and the large-scale human sacrifices associated with it, in terms of the material expression of divine authority, be it individual-centered [10] or of a repressive state [18]. This pyramid was burned down and its façade covered with a platform not too long after its construction. Millon interprets this event as the end of what had been a continuous reign of powerful rulers ([19]: 112). In addition, the existence of an early, centralized government seems to be supported by the following facts:

- During the city’s ascendency there were rapid, large-scale demographic shifts in the Valley of Mexico, with most of its population eventually resettling in Teotihuacan [7]. Millon suggests that these migrations must have been a deliberate policy that was enforced for the political advantage of keeping most of the Valley of Mexico’s population under direct control in the city ([20]: 103), with the rulers using force where necessary [21], [22].
- The rapid increase of population in Teotihuacan was accompanied by the construction of impressive public structures, including the three main pyramids. For Millon, the expenditures of energy manifest in the pyramids and the Street of the Dead are dramatic demonstrations of the exercise of power during a time of strong rulers ([21]: 25). Coe and Koontz concur that the pyramids attest the immense power of the early Teotihuacan hierarchy to call up corvée labor ([12]: 109).
- This construction of the city’s extensive ceremonial center, as well as of all other quarters of Teotihuacan, were realized in alignment with a specific orientation, namely around 15° east of true north. This suggests a high degree of planning, which in turn implies centralized control ([23]: 226). For instance, Cowgill interprets the alignment of the whole city as another sign of early strength of the central authority, which suggests the relative weakness of intermediate social units, such as large lineages ([8]: 155). In addition, several authors have argued that a substantial part of the city was built using a standard unit of measurement, which has been taken to imply the existence of a strong overarching central authority that could override local interests [24].
- One prominent theme of mural painting during the city’s later periods is the depiction of processions of richly attired persons, often armed with shields, arrows and knifes, and sometimes having bloody hearts impaled on them. This kind of imagery has been related to coercive violence and social status, and has been interpreted in terms of powerful military orders and the large-scale practice of human sacrifice [25], [26].
- Hundreds of human burials have been discovered in the Pyramid of the Sun, the Pyramid of the Moon, and the Temple of the Feathered Serpent. Many of the burials found in the Pyramid of the Sun were of infants and children [27]. By the time of the Aztecs there was a tradition of sacrificing children as an offering to the rain god, but we do not know if this was the case here. There is evidence that the burials in the Temple of the Feathered Serpent had militaristic associations [10]. Although their cause of death is not evident, the fact that their forearms crossed behind their bodies as if they had been bound at the wrists suggests that their sacrifice was not voluntary [28]. More compelling evidence for a practice of fighting wars to gain captives comes from the Pyramid of the Moon. Given that most of the people buried there were not originally from Teotihuacan [29], and some had been decapitated and deposited casually without regalia or offerings [30], they are likely to have been sacrificed captives. These findings support the hypothesis of a centralized political hierarchy at Teotihuacan because the origins of human sacrifice have been associated with the origins of elites in Mesoamerica [17].

### Evidence for Collective Government

Yet none of the above points is decisive with regard to the existence of centralized rulership, especially given the prominent lack of more direct archaeological evidence of the kind that is sometimes found in the context of Mesoamerican cities, such as supreme rulers’ tombs, royal portraiture, dynastic stele and inscriptions. Although differences in social status can be detected in Teotihuacan, for example in terms of burial practices and access to resources [31], these differences are neither very remarkable nor clearly localized. Moreover, the evidence cited above is consistent with alternative explanations.

- The wholesale emigration from other contemporary regional centers and the notably accelerated early population growth at Teotihuacan coincided with eruptions of at least two volcanoes in the southern parts of the Basin of Mexico. Most importantly, it has been argued that the cataclysmic eruption of the Popocatépetl volcano, dated to have occurred during the mid-first century AD, was responsible for the displacement of some 50,000 people northwards to Teotihuacan [32]. Just around 50 years later, there was another, smaller eruption in the southern parts, this time from the Chichinautzin volcano, which further contributed to their depopulation [33]. These migrations could therefore have simply been caused by geological factors [34]. Later Aztec accounts of the city’s origins, as recorded by Spanish friars, talk about migrations but do not mention that they were enforced by Teotihuacan [35]. Indeed, they explicitly speak of several old and wise leaders of the new settlers being installed as rulers, thereby placing Teotihuacan in the classic Mesoamerican tradition of co-rulership [22]. If migrations were enforced by local rulers it is unlikely that migrant rulers would have been accorded such elevated status. These Aztec accounts could be rejected as nothing but myth were it not for the fact that many of Teotihuacan’s temple complexes were designed according to migrant traditions (e.g., from Tetimpa, which had been destroyed by the Popocatépetl eruption [32]). We also note that during later times the city’s population was highly mobile, ethnically diverse, and included several foreign enclaves. Many people were born outside the city and then immigrated [36]. There is no evidence that these relocations were involuntary. In addition, the variable acceptance of the city’s customs and artistic traditions in the foreign enclaves suggests that enculturation was based on individual agency rather than centrally enforced (e.g., in Tlailotlacan, a Zapotec enclave [37]). The more likely scenario is therefore that the city originated in the coming together of several disparate and desperate groups, which would have facilitated the creation of a governing coalition. This is consistent with the fact that analyses of the density of potsherds from Teotihuacan’s earliest periods indicate several regions of dense occupation, rather than a single center [38].
- Cowgill reviewed the evidence about Teotihuacan’s social organization and accepted that powerful rulers were implicated in the construction of the Feathered Serpent Pyramid, but stops short of generalizing this state of affairs to earlier periods ([8]: 156). However, the cessation of the construction of new monumental structures after around 250 AD and the desecration of the Feathered Serpent Pyramid soon thereafter, which are often interpreted as due to a political change from a time of powerful rulers to more collective institutions (e.g., [39]), should also be reevaluated. It is surely no coincidence that the eruption of the Xitle volcano in the southwestern part of the Basin of Mexico, which destroyed the large settlement of Cuicuilco, has been dated to around 245–315 AD [40]. Additionally, evidence that some kind of transition took place during that time is not limited to the monumental core of the city, but includes diverse termination rituals in neighborhood centers, such as in Teopancazco [41]. In other words, although political changes may have been involved in this transition, we should not ignore the widespread ecological and religious impact of yet another nearby eruption. Even the function of the Feathered Serpent Pyramid as a ruler’s seat of power can be questioned; others view it as a grandiose ritual stage managed by a group of priests for events that involved the whole community [42]. And if the pyramids mainly served a ritual function for the community, then they would be better considered as large-scale public goods on a continuum with the constructions of large-scale housing for most of the population [43]. It has been argued that the construction of these apartment compounds makes this period of Teotihuacan history the foremost manifestation of collective government in ancient Mesoamerica ([15]: 9), so it could be expected that there were some antecedents in the city’s history.
- It seems likely that Teotihuacan’s canonical orientation was originally a result of the cosmological observations made with the Pyramid of the Sun, while other principal constructions followed the Pyramid’s alignments [44]. The highly prevalent imitation of this orientation could therefore have resulted from external constraints to ensure the maximal use of available space in a crowded city and, most importantly, from the religious significance of alignment with the city’s principal axis, which probably represented a vertical *axis mundi* [45]. In other words, we need to look no further than the widely accepted idea that the people of Teotihuacan considered their city to be a sacred cosmogram and the center of the universe [9]; it is unlikely that coercion was needed to convince immigrants to construct their compounds so as to share in this cosmic power. The fact that even the more distant residential structures adhere to the canonical orientation implies that the notion of sacred space was not limited to the ceremonial center but included the city as a whole. This is consistent with the hypothesis that the designs of the city’s prominent religious structures, namely the Three-Temple Compound and *talud-tablero* architecture, first originated in a domestic context [32], indicating that there was no clear distinction between sacred and mundane spaces. It also matches the findings of a space syntax analysis of the ceremonial core and of several apartment compounds, which suggest that the community structure of the apartment compounds and the city’s organization at the larger civic scale reflect each other [46]. Moreover, it was found that, contrary to traditional assumptions about the grid-like regularity of Teotihuacan’s layout, most of its streets are in reality short and discontinuous rather than long and straight, and the actual layout therefore does not fit with expectations of having been designed by one centralized authority. The situation is no different at the scale of apartment compounds. During the Teotihuacan mapping project it was recognized that the internal arrangement of each apartment compound is unique, which suggests that these structures were not built by the state to a uniform plan, “and there is no special reason to think that they were owned by the state” ([6]: 221). More detailed investigations of the spatial layout of the apartment compounds has confirmed this impression of diversity [47]. Indeed, excavations of a compound has provided evidence that “all building activities, including planning and construction, were initiated by people solely within the compound and that no help or support in construction came from outside of the compound” ([48]: 102).
- In contrast to the explicit depictions of the gory details of war, bloody human sacrifice, and the humiliation of captives by all-powerful rulers, which can be found in the artistic traditions of other Mesoamerican states, in Teotihuacan there is not a single piece of art depicting the subjugation of one person by another person. Instead there is a striking emphasis of abstractness, multiple perspectives, impermanence, playfulness, diversity, personal anonymity, and collective values [13], [49], [50]. Indeed, the most explicit representations of violence are found in a couple of atypical mural paintings of animals. In a mural painting known as “The Mythological Animals” two feathered serpents seem to be attacked or confronted by other animals, including felines, canines, and birds. Another mural painting depicts a pair of wolves slaying a deer and extracting its heart. It has been argued that this was the elite’s metaphorical justification for the practice of human heart sacrifice [26], but no archaeological evidence for this practice has yet been found. It is also worth noting that some artistic evidence for militarism seems to be more closely related with religious themes. For example, although many of the people buried in the Temple of the Feathered Serpent were decorated with necklaces consisting of human jaws, most of these were actually finely crafted replicas made from seashell and stucco [28]. Similarly, many of the obsidian arrowheads that were found scattered around the skeletal remains were unfit for use in actual battle [10]. These replica and substitutions suggest a concern for symbolic and ritual practices rather than an interest in physical power per se.
- While it was common practice for other Mesoamerican civilizations to wage wars with the specific aim of capturing enemies for use in rituals based on human sacrifice, this practice may not have been present in Teotihuacan. For example, the persons buried in the Temple of the Feathered Serpent were dressed like people from Teotihuacan [10]. An analysis of oxygen-isotope ratios in skeletal phosphate of 41 of these individuals revealed that most men had lived in the city for a prolonged period before their death, some since childhood and others after having moved from several foreign locations; most women had also lived in the city all of their lives or had moved from there to a foreign location as adults [51]. Moreover, these sacrifices may have been unique events. So far there is no archaeological evidence for large-scale human sacrifice outside of the specific context of dedication rituals during the construction of these three pyramids. Even the indirect evidence is sparse. For instance, Teotihuacan did not have any dedicated I- or T-shaped ball courts, an otherwise prominent and ubiquitous structure in contemporary Mesoamerican cities typically associated with elites and human sacrifice.

Finally, the hypothesis of a coalition government helps to resolve some of the outstanding puzzles. Specifically, it explains why direct evidence of individual rulership remains elusive even after decades of concerted archaeological search. In addition, it helps to explain the otherwise mysterious fact that the people of Teotihuacan chose not to make much use of systematic writing on permanent media, even though they were familiar with the writing systems of the Zapotecs and Maya [52]. Evidence of notation is so sparse that it was long thought that writing was absent altogether, although it now seems that a rudimentary system of signs was present [53] (e.g., glyphs forming part of mural paintings). What explains this strange self-limitation? Given that, as Marcus [54] has suggested, one of the main uses the Maya, Zapotec, and other cultures made of their writing systems was to exhibit and glorify the exploits of their royal lineages, e.g. on public stele, the relative absence of such writing at Teotihuacan is indicative of the relative absence of such lineage royalty. Similarly, given that the first instance of Zapotec script we know about was used to record the name of a captive who was depicted with his heart cut out [17], the scarcity of writing (and the absence of such graphic depictions in the city’s arts) indicates that this kind of military subjugation was assigned little importance. In other words, we can make better sense of the relative absence of public writing if we hypothesize that the city’s government consisted of localized forms of social interaction. We will return to this point in the discussion.

### A Network of Three-Temple Complexes (TTCs)

**Figure 2:**
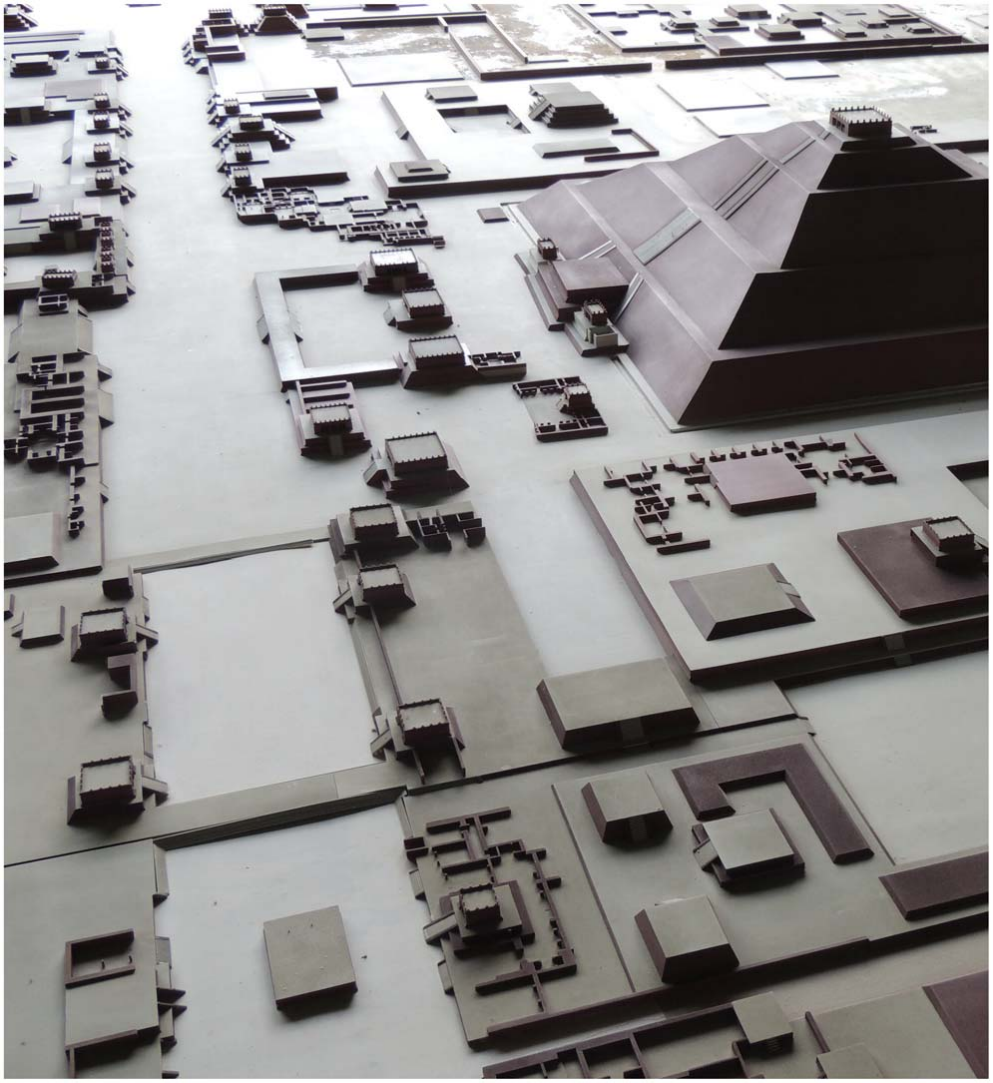
Architecture of a typical Three-Temple Complex (TTC). Central part of a mockup of the Street of the Dead (located in the Museo de Sitio de la Cultura Teotihuacana). The beginning of the plaza in front of the Pyramid of the Moon can be seen at the very top, while the Pyramid of the Sun is on the right. A typical TTC can be seen on the bottom right: three temples face each other around a square plaza, with the two equally sized temples flanking a larger temple in their middle. Another TTC can be seen in the center of the photo, with its plaza opening to the Street of the Dead. (Photo courtesy of Iliana Mendoza).

If Teotihuacan was governed by a coalition of representatives, how many representatives were there? We can derive a rough estimate by counting the number of administrative architectural units that were distributed throughout the early city, i.e. the so-called “Three-Temple Complex” (TTC) [9]. TTCs came in varying sizes and orientations, while generally conserving the arrangement of three temples on three sides of a rectangular plaza (Figure 2). While estimates of their number vary, there were at least 20 of them [55], [56]. It seems reasonable to assume that TTCs represented an administrative division of the city into neighborhoods during the city’s initial periods, although their exact role and relative status continue to be debated ([11]: 103–123). It is possible that the earliest TTCs constructed during the first century AD were originally elite houses from migrant groups that only subsequently acquired temple status [32]. Given their uneven distribution throughout the city, it is likely that they were not the only form of neighborhood center [11]; during later periods some of the larger “apartment” compounds may have served a similar role [14].

Many researchers interpret the TTCs as local manifestations of the state’s centralized power ([57]: 81–83), for which such neighborhood organizations would have provided an important intermediate organizational level ([6]: 225). On the other hand, Angulo [56] and Pasztory [55] see them as an indication that Teotihuacan emerged as a voluntary alliance between around twenty social units, possibly related clans, territorial units, or ethnic groups. Manzanilla ([58]: 59) agrees that TTCs are perhaps the earliest manifestation of the different groups that settled in the Teotihuacan Valley, following the volcanic eruptions of the first century AD. She argues that the purpose of the TTCs was to serve as centers of a redistributive circuit that organized economic surplus, in particular to pay full-time religious specialists and artisans [59]. This is in agreement with Paulinyi’s [50] extensive iconographic analysis, which suggests that the dominant layer of the city was composed of various groups of noble religious specialists, each based in their own temple, that shared political power in a distributed manner.

Possibly the representatives of these different groups were depicted in the famous Tassel Headdress murals, as described by Millon: “The procession of Tassel Headdress figures could represent the legendary founding of Teotihuacan by someone bearing the name of the Storm God. This figure might be leading twenty kin groups […] to the site of the later city. All of the kin groups could be depicted as participating in the city’s founding […]. There are problems with this interpretation, however, because it would not explain why all the figures wear headdresses symbolizing the Teotihuacan state” ([19]: 91). Of course, there are only problems with this interpretation because Millon assumes that Teotihuacan was founded by a legendary individual rather than by the twenty kin groups as a whole, that is, by one coalition. We note that the latter interpretation of these murals adds independent support for the model’s assumption of 22 relatively distinct social groups: even if one does not agree with the hypothesis that the twenty-something TTCs could have served as neighborhood centers during the city’s initial stages (e.g., [2]), the coalition government may have still consisted of roughly this number of social groups.

Nevertheless, although there exist these various strands of evidence that support the hypothesis of such a highly distributed collective government, the very possibility of this unusual type of political organization at the scale of a city often tends to be rejected on theoretical grounds. This is because the evolution from relatively egalitarian hunter-gatherer societies to “complex” societies is traditionally simply defined as the emergence of a centralized political hierarchy. Accordingly, it is commonly thought that the management of Teotihuacan necessitated a powerful centralized state apparatus ([57]: 79). To be fair, this kind of assumption is valid for many complex societies, and it also has a compelling theoretical justification: it is closely related to the problem of collective action, namely that the potential for selfish behavior would severely limit the possibilities of cooperation among a group of equals, thus resulting in the tragedy of the commons [60]. Accordingly, it seems that even if there weren’t any powerful individual rulers at Teotihuacan, there must have at least been a strong political class of elite bureaucrats. Blanton and Fargher, for example, have argued that there is an essential relationship between the extent of a society’s collectiveness and the extent of state involvement that is necessary in order to ensure cooperation ([61]: 29).

The possibility that a complex social system such as Teotihuacan could be successfully managed collectively in a self-organized manner, involving neither powerful leaders nor an extensive bureaucratic apparatus, therefore goes against ingrained assumptions of archaeology. An important part of the problem is the difficulty of even imagining the alternatives. However, Ostrom [62] has collected extensive empirical data which demonstrate that, at least in the case of smaller communities, shared resources can be effectively managed via self-organized government – thereby challenging skeptical sociological theories based on influential models such as the tragedy of the commons, the prisoner’s dilemma, and the logic of collective action. Thus, if Teotihuacan consisted of a diverse and polycentric network of communities, potentially represented by their TTCs, could this network not also have relied on a form of self-government rather than on a centralized coercive state? In the following we address this question by analyzing a minimalist mathematical model that makes it at least formally conceivable that Teotihuacan’s political consensus emerged from a distributed coalition that was not subject to one central institution.

### Overview of the Model

There is a growing interest in complementing traditional archaeological methods with computational tools, including by modeling social systems. One prominent approach is agent-based simulation in various grades of realism and predictive power [63], [64], while others prefer to directly model the nonlinear dynamics of social systems [65]. The lack of realism of the latter approach is offset by the generality of its explanatory insights. Another of its advantages is that we can use models that have already been systematically studied by other disciplines. For example, insights from the Hopfield neural network [66], which is isomorphic with the Ising spin model of statistical physics, are generalizable to distributed social systems [67]. Indeed, the cyberneticist McCulloch first introduced the concept of “heterarchy” to describe the organization of the nervous system, and it is from his work that archaeologists later adopted the concept to describe complex societies that were not (or at least not exclusively) hierarchically organized [68].

**Figure 3:**
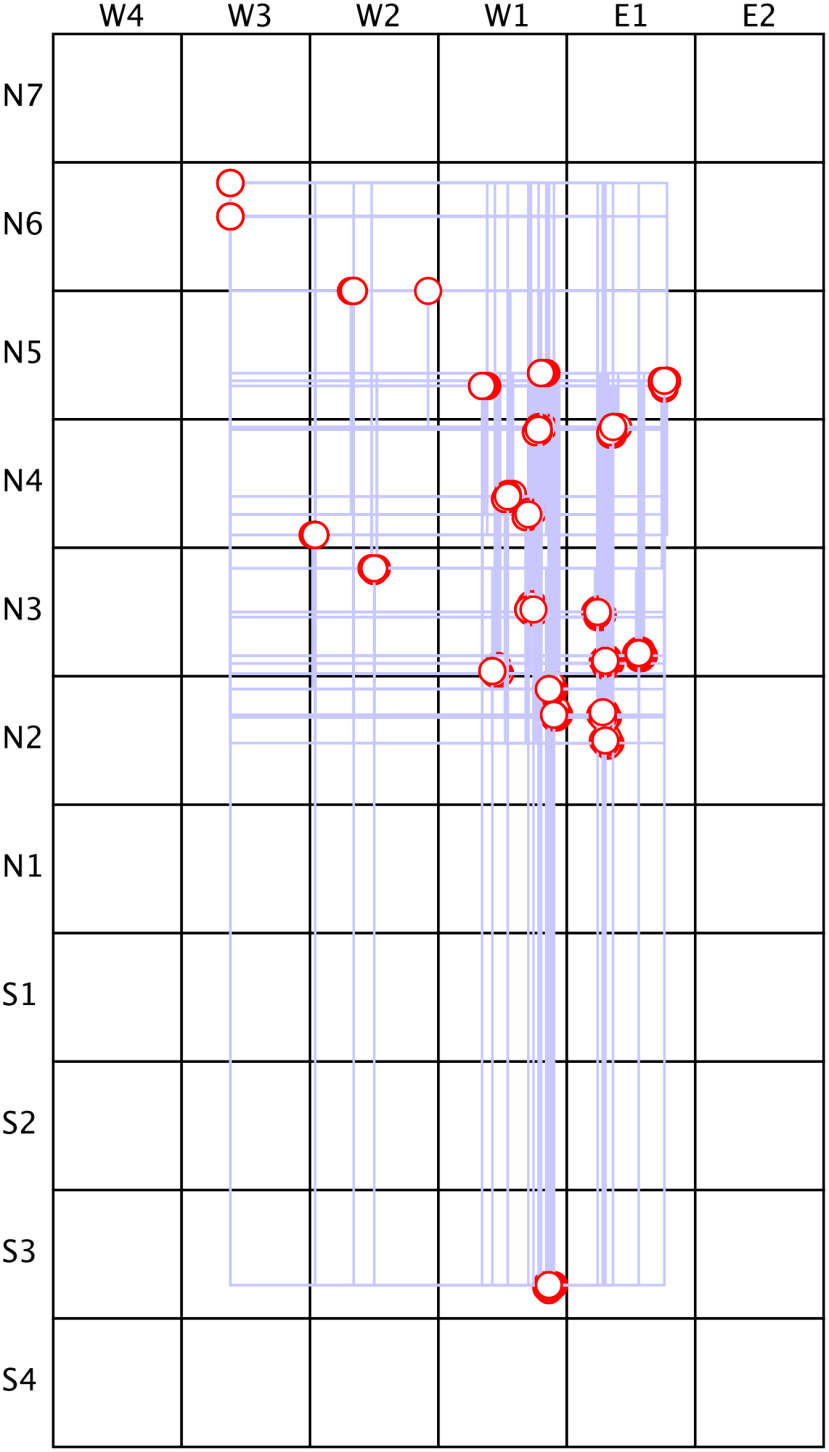
Spatial distribution of 22 Three-Temple Complexes (TTCs) of Teotihuacan. Only TTCs of an intermediate scale were selected as neighborhood centers. The Pyramid of the Sun, Pyramid of the Moon and known examples of smaller TTCs contained within excavated apartment compounds were excluded because they seem to belong to different spatial and temporal contexts. The shortest paths between any two TTCs are approximated as a rectangle because much of the city is densely occupied and roughly conforms to a grid-like pattern. Distances between TTCs were measured by hand on Millon’s [3] map. Minor measurement errors are visible, since the nodes do not overlap 100%. The effect of these distances on social constraints was incorporated into a pilot study, but not in the final model (see Methods section for details).

We use a variation of the Hopfield model based on the work by Watson and colleagues [67], [69]. Here we briefly present the basics of the model; further technical details are provided in the Methods section at the end of the paper. We assumed that the TTCs represent the city’s coalition government in its initial form, and identified 22 of them on Millon’s [3] map of Teotihuacan (Figure 3). To ensure that the best solutions require cooperation not only between neighborhood centers but also within neighborhoods, we assumed that there was a representative for each of the three temples of a TTC (*N* = 22*3 = 66). Representatives, also called agents, are modeled as nodes connected into a network. Each agent can be in one of two behavioral states (*s_i_* = +1/−1). Behaviors stand for a choice of social action (do-a/do-b), for example voting about public good options (vote-a/vote-b). At each time step a randomly selected agent adopts the behavior that maximizes its own utility, *u_i_*, defined as the weighted sum of its interactions with all the other members of the network.

With respect to the problem of collective action, this means the model adopts a worst-case scenario: any agent always chooses so as to satisfy the maximum number of its own social constraints, never putting the collective good before their personal good. It could be argued that this scenario is likely to be overly pessimistic, but it has the advantage that if our model succeeds in self-organizing a communal consensus under these conditions then it can be assumed that it will also do so in more pro-social scenarios.

Another possible worry is that an agent’s behavior is determined solely in relation to its current social network, thus neglecting the role of memory and intrinsic cognitive dynamics. In this respect the Hopfield network model is no different than most game theoretic approaches that derive behavior from simple payoff matrices [70]. Nevertheless, in spite of this simplifying assumption, they can still provide insights into actual public good dilemmas [71]. In addition, the inclusion of more complex cognitive dynamics seems to facilitate the emergence of an even wider variety of cooperative strategies [72], which leads us to expect that it might also help to mitigate the worst-case scenario assumed by our model. This is a prediction that could be investigated by future work.

In the Hopfield network model a connection weight *ω_ij_* represents the relative importance for agent *i* of satisfying the constraint that is posed by its interaction with agent *j*. In other words, agents will try to choose a behavioral state *s_i_* that ensures s_i_*s_j_*w_ij_>0 for as many connections as possible, but when satisfying different connections requires contrasting behaviors those with the stronger weights are given preference. Every weight *ω_ij_* is a combination of the weight specified by the original network topology, *ω^O^_ij_*, and the weight changes that accumulate as agents update the relative strengths of their connections, *ω^L^_ij_*. Agents adjust their relationships at a slower rate than updating their behavior; such a separation between slower and faster dynamical scales is a general condition for self-organization [73], [74]. In accordance with our assumption of the worst-case scenario, for each connection agents selfishly assess whether slightly increasing or decreasing its strength will increase their own utility and change it accordingly (by modifying the learned weight, *ω^L^_ij_*). Changes to an agent’s state *s_i_* depend on the original topology and the modifications that have taken place (i.e., *ω_ij_ = ω^O^_ij_*+*ω^L^_ij_*), but for purposes of analysis it is useful to know how well the network’s current state satisfies the original constraints alone (*ω^O^_ij_*).

As an illustrative example, we studied the self-organized coordination of voters into a global political consensus. All weights were set to positive values, which poses a consistent problem: configurations exist that allow every agent to satisfy all of their social constraints (i.e. all agents vote-a or all agents vote-b). However, this consistency does not mean that a globally optimal solution is easily found; that depends on the specific way in which the agents are connected and on their initial distribution of behavioral states. Weights between two nodes were symmetrical and no individual node was more powerful than all the rest. We investigated the impact of modularity (a network of neighborhoods) and nested hierarchies (a network of neighborhoods embedded within the city’s districts). The number of districts was equated with quadrants of the city [14], which is a conservative estimate (cf. [22]). More specifically, we consider two scenarios, i.e. neighborhoods and districts. In the *neighborhoods scenario* we assumed it was more crucial for representatives of each TTC to agree among each other (*ω_ij_* = 1) than to agree with representatives of other TTCs (*ω_ij_* = 0.01). In the *districts scenario*, we assumed the allegiance among a group of TTC’s representatives was maximal (*ω_ij_* = 1), as well as comparatively stronger to other representatives of the same district (*ω_ij_* = 0.02) when compared to other districts (*ω_ij_* = 0.006). For ease of comparison of utilities, the connection weights were selected such that the weight sum of the scenarios was equal.

Archaeologists and art historians are in agreement that integration of the various groups in Teotihuacan depended on the public promotion of a collective ideology, and that public ritual activity was foundational for the establishment of Teotihuacan. Indeed, shared rituals become especially important if we assume a collective government because it can ensure cooperation: “ritual provided the unifying force for peaceful coexistence among the different ethnic groups during the early years of the protocity” [56]. Ritual activity, like symbolic activity more generally, is primarily based on arbitrary cultural conventions. It is distinguished by conventions that do not seem to seem to have any immediate functional value, for example prohibitions against eating certain types of food on auspicious days. But the communal enactment of such non-ordinary behaviors is a transformative performance that brings the community together. Turner [75] concluded from his extensive research into tribal rituals that what is jointly enacted through them is a domain of the “uncommon sense” in which people are temporarily released from the pragmatic constraints of daily life. He famously characterized this liminal phase of the ritual process in terms of “anti-structure” in order to distinguish it from the structure imposed by mundane social relations [76].

We therefore modeled shared rituals as occasional interruptions to normal behavior, whereby the converged behaviors of all representatives are simultaneously replaced by arbitrary states (each behavior is randomly set to either +1 or −1). Each ritualized interruption is followed by the network’s convergence on another equilibrium, i.e., a normalization period of sufficient duration such that constraint-satisfying behaviors have been regained by the coalition and utilities remain constant once again. This mechanism of behavior randomization should not be misunderstood as isolating the individuals from each other in general; rather, it represents a temporary bracketing of their normal social constraints. As we will see below, it is in fact essential that most of the community takes part in this ritual process at the same time. And, as would be expected, in the long term these interruptions actually have the spontaneous effect of bringing the individuals closer together than before, thereby serving as a unifying force.

## Results

**Figure 4:**
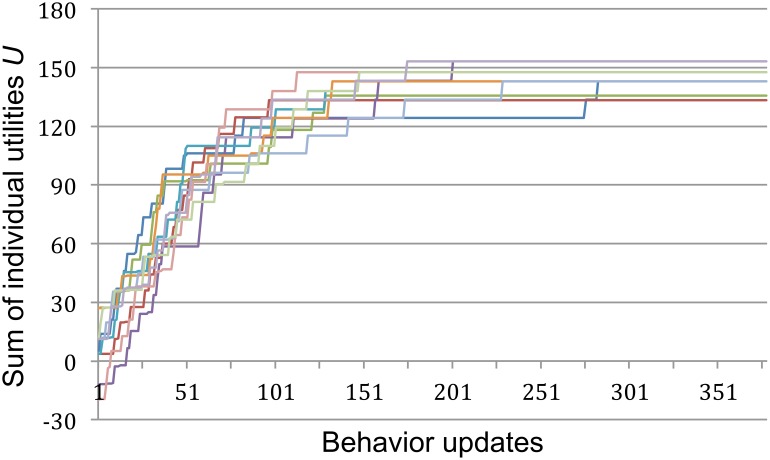
Ten examples of self-organized constraint satisfaction before self-optimization. During each behavior update a representative in the network is randomly selected, and allowed to adjust its behavior (to +1 or −1) if the new choice satisfies more constraints posed by its connections with all the others. Because a representative’s received utility is calculated as the weighted sum of its satisfied constraints, priority is given to satisfying more important (more weighty) connections. As representatives repeatedly optimize their choices, the network’s sum of utilities *U* increases. However, as shown by these 10 independent trajectories starting from arbitrary initial conditions, this weak form of self-organization typically becomes trapped in one of several suboptimal behavioral configurations (*U* = 173.58 for global optima). Only first 400 behavior updates shown; after that *U* stays the same until the end of the run in all cases.

Formally, there are two globally optimal behavioral configurations depending on the type of complete political consensus; either all agents vote for option ‘−1’ or all agents vote for option ‘1’ Given the original constraints of the neighborhoods scenario, either preferred outcome is equal to a utility sum of 173.58. However, it is rare for the network of agents to automatically converge on either of these two solutions – at least without rituals. In a sample of 200 independent random initializations and convergences, such a global consensus was never found (the highest utility sum was 166.02 while the average was 146.74 with a standard deviation of 8.19). This weak form of self-organization is a clear example of the problems faced by collective action in a heterarchical system, whereby the competing interests of selfishly and rationally behaving individuals prevent the emergence of more preferable solutions dependent on cooperation (Figure 4).

**Figure 5:**
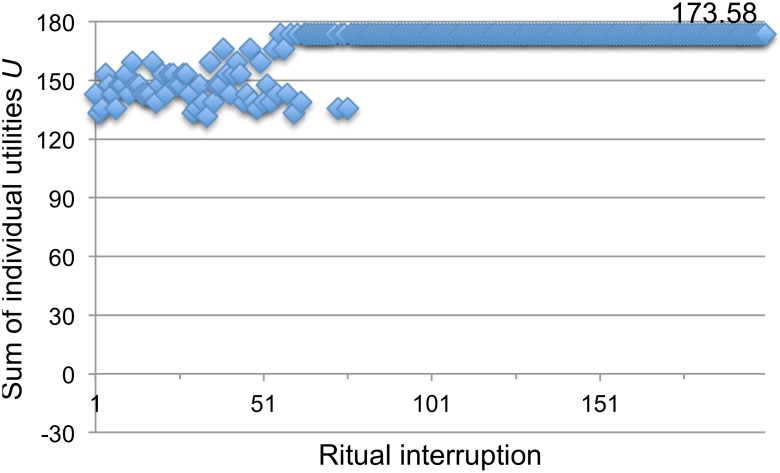
Sum of utilities reached after convergence during a series of 200 ritual interruptions. In contrast to Figure 4, convergences happen in sequence rather than independently, and only the final sum of utility is plotted (behavioral updates leading to convergence are not shown). During a ritual interruption all behavioral states are reset to arbitrary states, as before, but agents’ changes to their social connection weights are retained. To allow comparison with Figure 4, we show the sum of utilities *U* based on the original network’s constraints only. Self-optimization is clearly visible: the network is quickly and spontaneously transformed such that updates of behavior, despite being selfish and rational, consistently converge on a globally optimal configuration (*U* = 173.58).

However, the results are rather different when the randomizations and re-convergences are treated as events of the same social system, namely as a sequence of community rituals. Specifically, behaviors were occasionally set to arbitrary behavioral choices and allowed to converge to an equilibrium, as before, but this time agents’ changes to their connections were allowed to accumulate over 200 such ritual interruptions. Under these conditions agents managed to reach a complete consensus already after the 56^th^ ritual, and from the 77^th^ ritual onwards this globally optimal solution to the original problem was consistently reached from every arbitrary configuration of behaviors (Figure 5). This is a strong form of self-organization that we refer to as self-optimization.

**Figure 6:**
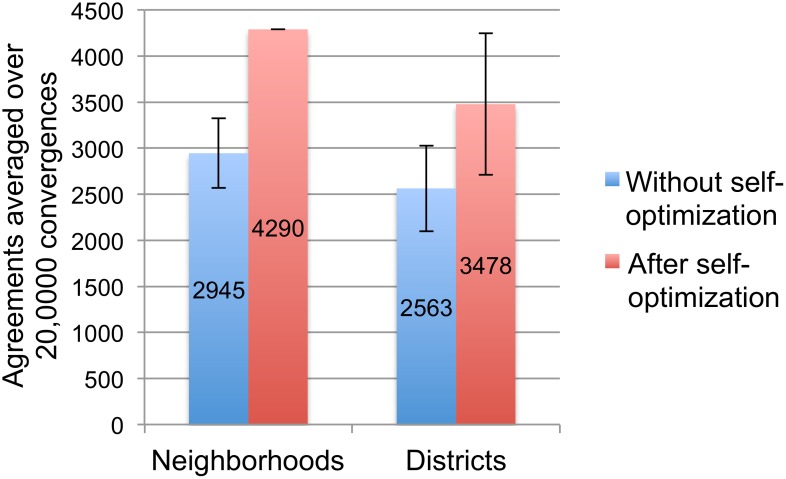
Comparison of the extent of cooperation in four variations of the Teotihuacan network model. We compared cooperation in two respects: As a function of self-optimization, i.e. whether connection changes were preserved over the preceding 200 ritualized perturbations or not (red versus blue bars). And as a function of social organization, i.e. whether the network is divided only into 22 neighborhoods or also additionally into four larger-scale districts (Neighborhoods versus Districts). For each case we assessed the outcomes of 20,000 independent convergences. The bars show the average number of agreements reached by the social network given the constraints of its original organization (4290 agreements equal complete consensus). Error bars represent one standard deviation.

We compared cooperation in the neighborhoods scenario with the districts scenario, in which the 22 neighborhoods are categorized into the four districts of early Teotihuacan. We measured cooperation as the total number of agreements between agents’ behavioral states that satisfy the constraints of their connections; an agreement is counted whenever s_i_*s_j_*w_ij_>0. The maximum number of agreements is 4290 ( = 66*66 connections–66 self-connections). To assess the lasting impact of network self-optimization we compared the average number of agreements for both scenarios in 100 separate runs reach. As before, a run consisted of 200 converges to equilibrium. Thus, we compared a total of 20,000 independent convergences of the original topology with 20,000 independent convergences reached after self-optimization had already been completed (Figure 6). For purposes of comparison, the number of agreements reached by the behaviors of the modified social network is calculated only with respect to the original weight matrix, *ω^O^_ij_*.

In the neighborhoods scenario, agents connected in the original network rarely managed to resolve all of their constraints without ritual interruption. Out of the 20,000 independent convergences only 64 reached the 4290 agreements needed for a complete consensus. Most frequent were medium levels of cooperation, with an average of 2945 agreements. This problematic state of affairs was completely transformed by self-optimization, which for each of the 100 runs consisted of a series of 200 ritual interruptions of agents’ behaviors while their changes to connections were preserved. Of the 20,000 independent convergences *after* self-optimization only 7 failed to realize the maximum number of agreements. Remarkably, this outcome of self-optimization does not depend on the network encountering and remembering globally optimal configurations. In most cases, the network had never even visited such an optimum prior to self-optimization, thereby showing that the process does not depend on *a priori* knowledge of the structure of the underlying problem space.

In the districts scenario, agents managed to resolve their constraints even less often without self-optimization. Out of 20,000 independent convergences only 53 realized a complete consensus. Most frequently there were medium levels of cooperation with an average of 2563 agreements. Conditions were improved after self-optimization, even if not to the extent of the neighborhoods scenario: the maximum number of 4290 agreements was reached 7544 times, while the average number of agreements was 3478. Self-optimization therefore frequently resulted in network architectures significantly more capable of cooperation, but the outcome was more variable. On many occasions the process converged on suboptimal configurations, even if optimal or at least better solutions had previously been encountered. Suboptimal solutions typically consisted in one or more of the network’s subsections, e.g. whole neighborhoods and/or whole districts, resisting behavioral alignment with the global consensus.

Another important issue to consider is the average amount of time that is typically required to reach a global behavioral consensus and to complete the process of structural self-optimization. The time taken for structural convergence will be briefly described in the discussion section below. Here we focus on the time scale of individual behaviors. We calculated the number of behavior updates taken to reach the final utility value for each of the 200 behavioral convergences of each of the 100 experimental runs. In the case of the neighborhoods scenario we found that before self-optimization there were on average 225 behavior updates (with Standard Deviation, SD = 82) before reaching convergence, whereas after self-optimization an average of 264 updates (SD = 82) were needed. In the case of the districts scenario an average of 225 (SD = 81) and 269 (SD = 86) updates were required, respectively.

In both scenarios significantly more updates were taken after self-optimization, which is to be expected given that the aim of self-optimization is precisely to avoid premature convergences on only partially optimal behavioral consensuses. Yet this represents only around 3 to 4 behavioral updates per individual representative until system convergence, given that the network consists of 66 representatives. We note that that this is again a worst-case scenario because it is the time taken to reach a new consensus after completely scrambling any previously existing behavioral configuration. In everyday situations the impact of ritualized challenges to the social equilibrium is likely to be smaller and recovery of normal consensus correspondingly faster. However, we emphasize that it is difficult to give more precise estimates about the time to convergence in real time, since the model is too abstract to specify the duration of a unit of behavior.

## Discussion

The model is in agreement with the traditional assumption that collective action is faced by serious problems without centralized hierarchical control, but it also clearly shows that spontaneous cooperation is feasible without it. At least in principle, there is no necessity to assume the existence of a lineage of powerful rulers to explain the origins of Teotihuacan. A coalition government, perhaps as expressed by the TTCs, could have been present from its beginnings. Moreover, given that the model requires certain conditions in order for the process of self-optimization to be effective allows us to put forward the hypothesis that similar conditions were also present at Teotihuacan. The model therefore has predictive power that can inform archaeological investigation. In the following we explore some of the implications that could be topics of future research.

**Figure 7:**
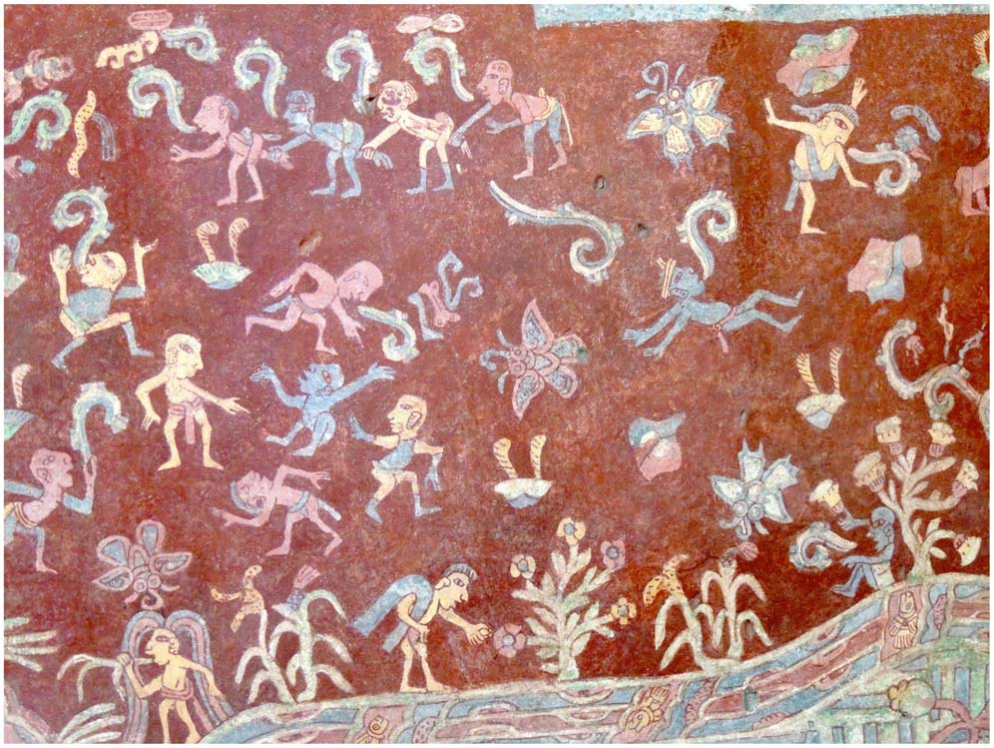
Mural painting located in the Tepantitla apartment compound. This is a representative section of a much more extensive scene showing a large number of modestly dressed people who are engaged in a variety of unusual activities. Most of them do not seem to be engaged in the mundane activities imposed by the needs of daily life. Some of the people’s interactions with plants and flowers have been interpreted in terms of the consumption of psychoactive substances (see, e.g., on the bottom left where a person is eating a plant while rainbow-like streams are flowing from his head). Scenes such as these are indicative of large-scale communal rituals or events at Teotihuacan. (Photo courtesy of Iliana Mendoza).

We take it for granted that individuals will behave and change their social ties in a way that primarily benefits themselves (although in fact this assumption is not necessary for this self-optimization [67]). But the peculiar requirement that the behaviors of the social system as a whole are occasionally interrupted makes a more specific prediction. We can therefore hypothesize that the ritual calendar of Teotihuacan included infrequent, large-scale events during which normal social activity and norms were suspended. Symbolic behavior largely consists of arbitrary customs, and it therefore makes a suitable candidate for the process of systemic interruption by means of behavior randomization in our model. Ritual intoxication, which was a common practice in ancient Mesoamerica [77], could have further enhanced the perturbation. It is suggestive that mural paintings in the apartment compounds of Tepantitla [78] and Atetelco [42] depict large-scale social events that include intoxication (Figure 7).

If we assume that such ritual events took place twice a year, for example at the start of the rainy and dry seasons, then the structural self-optimization exhibited by our model would have always been completed in less than 100 years. This is consistent with the time it took for Teotihuacan to grow from a small town to its full extent as a metropolis. For example, the self-optimization of the coalition network to a globally optimal configuration shown in Figure 5 would already have been completed after only 38 years, which presumably is equivalent to one or two generations of representatives. Small subsequent adjustments to this optimal configuration could probably happen much faster, thus making the coalition quite adaptable. Future work could formally investigate the network’s adaptability to continuously changing optimality conditions.

Fittingly, there are many large open spaces in the city’s ceremonial core that could have accommodated such ritual events. Excavations at the Pyramid of the Moon found no evidence for durable structures on top of its successive construction stages, which suggests that they were elevated platforms for large public rituals that could be witnessed at ground level [39]. This is in contrast to the common Mesoamerican tradition of building small and enclosed temples on top of the pyramids, whose indoor activities would have been visible only to a few elites who were granted access. In particular, the model supports a religious interpretation of the enormous building complex known as La Cuidadela, which contains the Feathered Serpent Pyramid and a huge open plaza. Archaeologists Gómez-Chávez and Gazzola have argued that, rather than being the political base of a powerful ruler, La Cuidadela functioned as a grandiose ritual stage that incorporated the yearly flooding brought on by the rainy season and in which all of society participated ([42]: 238).

The results also speak to another aspect of the problem of Teotihuacan’s origins. It is difficult to imagine how individual rulers and/or bureaucrats could have intentionally designed and managed the rapidly growing city, especially given that there was no existing tradition of large-scale urbanism to draw on. However, our model makes no assumptions regarding the explicit knowledge and managerial capacities of Teotihuacan’s representatives, since it is a self-managing system whose adaptive order can spontaneously emerge from purely localized social interactions given the right kinds of conditions.

It goes without saying that the possibility of a coalition government fits with Teotihuacan’s unique artistic tradition and the notable absence of public dynastic monuments [43]. The results also suggest an explanation of another one of Teotihuacan’s unique cultural traits that has remained a mystery so far, namely the fact that there was not much interest in emulating Maya or Zapotec literacy. This raises questions about the nature of a society that largely dispensed with complex writing on a voluntary basis [79]. To be sure, in the absence of great individual rulers, there was no need to record dynastic information. But it remains difficult to imagine how a city of this complexity could have functioned without relying on extensive record keeping, especially if it had a collective government that had to ensure the cooperation of its members. Such a corporate strategy has been associated with an increased, rather than reduced, need for bureaucracy [61]. Of course, it remains possible that extensive record keeping was done on perishable media that has had little chance of being preserved over the centuries. Yet our results suggest another possibility, namely that even record keeping on impermanent media was a rather minimal practice. This is because in our model the choices of representatives are guided by immediate concerns only, without any explicit consideration of past behaviors, future plans, or rules of reciprocity and punishment. To be sure, this assumes an overly extreme lack of historicity, but it nevertheless suggests that it is possible for a government to be collective without a powerful bureaucracy, and by extension, with little need for extensive record keeping. In addition to the classes of ruler-controlled centralized states and bureaucracy-controlled collective states there may also have been a class of ritually self-optimized collective states whose unique social organization has so far largely gone unnoticed by archaeology due to a lack of both individual personality cults and extensive record keeping.

Finally, we can also draw insights from the model regarding what may have caused the downfall of Teotihuacan. The districts scenario showed that ritualized self-optimization struggles to develop its full potential when sections of the network are too independent, so the model predicts that parts of the city became increasingly autonomous before its end. Indeed, this process is reflected in the archaeological record. Starting in the city’s middle period, most inhabitants formed small communities that lived in self-contained apartment compounds. These walled compounds increased their autonomy towards the city’s end, as indicated by controlled access points and direct trade routes to foreign partners. In later periods social divisions at the district-level of the city also became more prominent, which may have further fractured the city [14]. Given the crucial role played by system-wide ritual interruptions in this model, we speculate that Teotihuacan’s end was also precipitated by a decrease in the relative importance of citywide rituals compared to compound rituals.

## Methods

In this final section we provide a more detailed description of the model and the design choices that were made in its creation.

### Mathematical Details

We follow Watson and colleagues [67], [69], [80] in using the network architecture first proposed by Hopfield [66]. Each agent can adopt one of two discrete behavioral states, *s_i_* = +1/−1, which stands for a choice of action (do-a/do-b). We use an asynchronous updating rule, which means that for each point in time *t* an agent is randomly selected to update its behavior for time step *t*+1. The selected agent will adopt the behavior that maximizes its own utility, *u_i_*, which is defined as the weighted sum of its interactions with other agents,
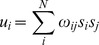
where the connection weight *ω_ij_* represents the importance for agent *i* of satisfying the constraint posed by its interaction with agent *j*. The more positive *ω_ij_*, the more important it is for agent *i* to imitate the behavior of agent *j* (such that both do-a or both do-b), while the more negative *ω_ij_*, the more important it is for agent *i* to complement the behavior of agent *j* (such that agent *i* and agent *j* either do-a and do-b, or do-b and do-a, respectively).

We distinguish two aspects of *ω_ij_*. On the one hand we specify and retain the original problem space or network topology, *ω^O^_ij_*, while on the other hand we keep track of the changes that accumulate as agents selfishly update the weights of their interactions, *ω^L^_ij_*. These weight changes are equivalent to simplified Hebbian learning in Hopfield neural networks [80]. While the original weights *ω^O^* are static throughout a run, the learned weights *ω^L^* depend on the current time step, and both make up the current weights of the network:
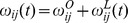



While the update of an agent’s state *s_i_* is determined by how it evaluates its modified connections, *ω*, it is possible to determine how this state affects the network’s ability to satisfy the constraints of the original weight space *ω^O^*. The sum of utilities given the original problem space is calculated as follows:
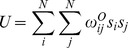



We assume that agents are selfish and rational, which means that for each social connection they systematically assess whether increasing or decreasing its strength will increase their individual utility. In other words, the changes implied by both Δω*_ij_* = +*r* and Δω*_ij_* = –*r* are considered, and whichever will increase individual utility the most is accepted. We fixed the learning rate *r* to be the same for all of the experiments (*r* = 0.0015). If neither change provides an increase the connection remains unchanged. For convenience a change is only applied once at the end of a convergence. Similar results would be obtained if a smaller learning rate were applied continuously as long as the system spends most of its time in a converged state [69]. Accordingly:
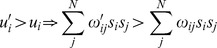



It may be questioned whether real agents can behave as rationally as this, but the assumption of perfect rationality has little impact on the overall dynamics of the social network. Similar effects can be obtained by assuming that agents generally behave in a habitual manner, such that the propensity of agent *i* to imitate (or instead to complement) agent *j*’s behavior will be enhanced if agent *i* is currently imitating (or complementing) agent *j*’s behavior [67]. Given that social connections cannot be modified infinitely, we assume that weights are limited to the range [–1, 1] by a linear threshold function *θ* such that if *x*>1 then *θ*(*x*) = 1 or if *x*<−1 then *θ*(*x*) = −1. In other words:
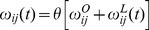
where



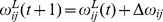



Following Hopfield’s original proposal [66] we also enforce that the connection weights are initialized symmetrically, i.e. *ω_ij_* = *ω_ji_*. This ensures that the network will eventually settle into a fixed-point attractor. Allowing asymmetrical connections (i.e., *ω_ij_*≠*ω_ji_*), for example such that agent *i*’s next behavioral state is more dependent on the behavior of agent *j* than *vice versa*, does not alter the general fact that selfish updates of connections resemble Hebbian learning [69]. However, a network that includes strongly asymmetrical connections allows for a greater variety of attractor types; future work should investigate their effects. We also ignore self-connections, i.e. *ω_ii_* = 0. Their inclusion would simply have the effect of increasing the system’s total utility *U* by a constant amount, since an agent’s behavior will always be identical with its own behavior.

Following Watson and colleagues, a new set of random behavioral states, *R* = [−1|1]*^N^*, was assigned every *τ* time steps; we set *τ* = e*N* ln*N*, where *N* = 66. We set the number of behavior randomizations to 200, which was sufficient to allow self-optimization to occur in most cases.

### Spatial Considerations

We did not modulate the network’s strengths of connections in relation to the spatial distance of the TTCs from each other (i.e., as shown in Figure 3), because a pilot study of a 22-node spatially embedded network, in which strength of connection was inversely proportional to distance, did not reveal significant effects of space compared to a non-spatial network. It is possible to attribute this irrelevance of spatial distance to the spatial distribution of TTCs. When we analyzed the frequency of distances, we found that most TTCs are very close to each other with only a few long-distance outliers. In other words, if we take spatial proximity as a marker of the strength of mutual interconnectivity, then a network of 22 spatially embedded TTCs has more or less the same connectivity as a fully connected 22-node network. The absence of a scale-free distribution of connectivity is interesting in itself, because archeologists typically view scale-free networks as an indicator of social hierarchy [81].

Archaeologically speaking, choosing to investigate a non-spatial network of neighborhood representatives also makes sense if all representatives met in a single location to discuss and vote on policies, which seems to be a reasonable assumption in the case of a Teotihuacan coalition. For example, a systematic analysis of surface remains has indicated that during the city’s later years the interests of the various neighborhoods might have been represented in apartment compounds that were co-located in a large forum known as the Great Compound [82], which is situated at the southern part of the Street of the Dead across from the Feathered Serpent Pyramid. Another promising location for a unified seat of the coalition government is a compound known as Xalla [83]. This alternative hypothesis is currently being investigated via excavations conducted by Manzanilla as part of the project “Teotihuacan: Elite y gobierno”.

Further choices had to be made regarding how to design the districts scenario. Evidence exists that during later periods of the city the neighborhood centers were organized into larger districts, although retaining a strongly heterarchical emphasis [22]: up to 45% of apartment compounds were comparable in type across the city, overshadowing neighborhood membership, religious affiliation, and status [84]. Manzanilla [14] has argued that during this time the city was divided into four separate districts, that is, from North to South along the Street of the Dead and from West to East via the Great Compound and the Feathered Serpent Pyramid. There are indications that during earlier periods the West-East axis was located further north in the city, across the Pyramid of the Sun ([58]; R. Cabrera Castro, personal comm.). Since we are interested in modeling the initial period of Teotihuacan, it is this earlier West-East axis that we used in our model to define the four districts of neighborhoods. We therefore ended up with the following districts: Northwest (10 TTCs), Northeast (2 TTCs), Southwest (5 TTCs), and Southeast (5 TTCs). Future modeling work could look at other ways of carving up the city’s space and at changing the number of social units participating in the network. Nevertheless, due to the generality of the self-optimizing process we expect that at least qualitatively similar results can be obtained under a variety of conditions.

### The Self-Optimization Process

Further work is required to fully understand the mathematical basis and scope of the self-optimization process [85], but its effects should be noticeable in any heterarchically organized system that satisfies the following conditions:

- Agents try to optimize their own situation by rapidly adjusting their behavior in relation to what others are doing,
- Agents try to optimize their own situation by slowly adjusting their social relations in relation to what others are doing, and
- Agents, the more the better, occasionally jointly change their behavior in some arbitrary manner.

Mathematically speaking, condition (a) causes the social system to quickly converge on an optimum, although in most cases it will only be a locally optimal one, (b) causes the system to form an associative “memory” of the already visited optima, and (c) causes the system to visit different kinds of optima over longer periods of time, thereby causing the associative memory to become generalized in such a manner that better optima are more easily encountered [69].

It is condition (c) that is of special interest. Selfish updates of connections after converging on only one attractor would not be adaptive. Given that in most complex networks the number of suboptimal attractors by far outnumbers more optimal configurations, it is most likely that the network would become trapped in a local optimum, and the adjustments of connections would therefore simply have the undesirable effect of even further reinforcing the suboptimal attractor. However, the outcome is quite different when we occasionally randomly reset or otherwise perturb the system, followed by periods of sufficient time to reach convergence. If the perturbations are sufficiently powerful then the system will start itinerating over its different attractors, exploring the attractors that are possible to reach from different initial conditions. Watson and colleagues discovered that under these conditions the system self-optimizes its connections such that better configurations are more likely to be visited [69]. There are two aspects to this self-optimization process.

#### Enhanced recall

It has been shown that in this type of network better (i.e., higher or deeper, depending on the sign used to define utility) attractors have larger basins of attraction [86]. This entails that the better attractors, although numerically less common than suboptimal optima, will be visited relatively more frequently in a long sequence of restarts. The Hebbian-style learning implemented by the changes to connections will therefore tend to reinforce the better attractors more strongly, which means that their likelihood of visitation is further increased until only the best attractors remain.

#### Generalization

But how do we explain that the network frequently converges on globally optimal solutions, which it had never visited before, and much faster than should normally be expected? This surprising effect can be accounted for by the fact that the network starts to generalize over all of the attractor configurations that it has already reinforced. This generalization is a well-known property of trained Hopfield networks, which exhibit a type of associative memory [87], except that in this case the set of training patterns is the set of visited attractors of the network itself. One way to think about this is to consider that when the visited attractor configurations of the network are decomposable into shared features, for example due to modularity, the selfish modifications of connections can lead to the enhancement of basins of attraction of not-yet visited attractors consisting of better combinations of those same component features [69].

Accordingly, the model provides a genuinely system-level explanation of collective government: globally optimal architectures tend to be spontaneously arranged by the agents even though each agent is behaving selfishly and even though no agent has knowledge about the structure of the overall problem space. Adaptive management of the social system as a whole emerges out of the local interactions among members of the social network. It is noteworthy that this process is based on formal principles that do not depend on the specifics of the current model.

The very possibility that a complex social system as a whole can self-organize its governmental functions successfully – without relying on the usual mechanisms of centralized top-down control, without depending on any explicit representation or knowledge of the solutions, and more generally while avoiding the extremes of social stratification – is something to keep in mind when addressing today’s seemingly insurmountable global crises.
